# Measurement Science for Prognostics and Health Management for Smart Manufacturing Systems: Key Findings from a Roadmapping Workshop

**Published:** 2015

**Authors:** Brian A. Weiss, Gregory Vogl, Moneer Helu, Guixiu Qiao, Joan Pellegrino, Mauricio Justiniano, Anand Raghunathan

**Affiliations:** 1,2,3,4 National Institute of Standards and Technology (NIST), Gaithersburg, Maryland, 20899, USA; 5,6,7 Energetics Incorporated, Columbia, Maryland, 21046, USA

## Abstract

The National Institute of Standards and Technology (NIST) hosted the *Roadmapping Workshop – Measurement Science for Prognostics and Health Management for Smart Manufacturing Systems (PHM4SMS)* in Fall 2014 to discuss the needs and priorities of stakeholders in the PHM4SMS technology area. The workshop brought together over 70 members of the PHM community. The attendees included representatives from small, medium, and large manufacturers; technology developers and integrators; academic researchers; government organizations; trade associations; and standards bodies. The attendees discussed the current and anticipated measurement science challenges to advance PHM methods and techniques for smart manufacturing systems; the associated research and development needed to implement condition monitoring, diagnostic, and prognostic technologies within manufacturing environments; and the priorities to meet the needs of PHM in manufacturing.

This paper will summarize the key findings of this workshop, and present some of the critical measurement science challenges and corresponding roadmaps, i.e., suggested courses of action, to advance PHM for manufacturing. Milestones and targeted capabilities will be presented for each roadmap across three areas: *PHM Manufacturing Process Techniques; PHM Performance Assessment*; and *PHM Infrastructure – Hardware, Software, and Integration*. An analysis of these roadmaps and crosscutting themes seen across the breakout sessions is also discussed.

## 1. Introduction

The National Institute of Standards and Technology (NIST) is a research agency with the United States (U.S.) Department of Commerce that develops measurement science to advance innovative and emerging technologies to increase U.S. industry’s competitiveness on a global scale. One specific area of NIST research is focused on Prognostics and Health Management for Smart Manufacturing Systems (PHM4SMS). To that end, it is critical for the NIST-PHM4SMS project team to understand the needs of its stakeholder community to develop and evolve the project’s research plan, accordingly. A *Roadmapping Workshop – Measurement Science for Prognostics and Health Management for Smart Manufacturing Systems* was hosted by NIST on November 19^th^ and 20^th^, 2014 to discuss the needs and priorities of stakeholders in the PHM4SMS technology area. The workshop brought together over 70 members of the PHM community including representatives from small, medium, and large manufacturers; technology developers and integrators; academic researchers; government organizations; trade associations; and standards bodies. The attendees discussed the current and anticipated measurement science challenges that they felt the PHM community should address to advance PHM methods and techniques for smart manufacturing systems. Attendees also described the associated research and development (R&D) needs that are hindering the advancement and implementation of condition monitoring, diagnostic, and prognostic technologies within manufacturing environments. Finally, attendees identified the priorities and next steps to meet the needs of PHM in manufacturing.

This paper begins by offering background on NIST’s efforts in Smart Manufacturing and PHM in Section 2. Section 3 summarizes the key findings of the workshop including highlights from the panel discussions and breakout sessions. The breakouts focused the participants in three areas: *PHM Manufacturing Process Techniques; PHM Performance Assessment*; and *PHM Infrastructure – Hardware, Software, and Integration*. For each breakout, participants identified the area’s goals, desired capabilities, challenges and barriers to developing these capabilities, and specific roadmaps with milestones and targets to achieve these goals and capabilities. Section 3 also highlights some of the crosscutting themes that emerged throughout the workshop. Section 4 concludes with a discussion of the NIST-PHM4SMS team’s existing research plans.

## 2. Background

### 2.1. Smart Manufacturing Systems (SMS)

NIST’s mission is to promote U.S. competitiveness across many technological areas including manufacturing. Smart Manufacturing has been identified by numerous U.S. leadership organizations (including the Executive Office of the President) as a necessity for U.S. manufacturers to increase their global competitiveness (Manyika, Sinclair, Dobbs, Strube, Rassey, Mischke, Remes, Roxburgh, George, O’Halloran & Ramaswamy, 2012) (PCAST, 2012) (PCAST, 2014). Smart Manufacturing Systems (SMS) are the synthesis and integration of advanced physical and virtual technologies to enable innovative processes and enhance existing methods. SMS includes the convergence of information and communication technologies with a range of sophisticated and emerging capabilities in a wide range of domains including sensing, automation, machining, robotics, and additive manufacturing. The effective and efficient use, and integration of these technologies is promoting manufacturing growth by enabling manufacturers to increase their productivity, quality, and safety, while reducing their costs and waste (Bernaden, 2012).

NIST has developed a suite of Smart Manufacturing programs (including robotics and additive manufacturing) to address the measurement science challenges faced by manufacturers who are actively looking to grow and/or enhance their operations. One of the programs is the Smart Manufacturing Operations Planning and Control (SMOPAC) program which is designed to tackle technological and integration challenges posed at the factory level.

PHM is a critical part of Smart Manufacturing. PHM may ultimately enable a machine or system to self-diagnose and self-heal with enough intelligence to be both aware of its current health and make an appropriate decision given both its state and goals. Presently, condition-monitoring, diagnostic, and prognostic techniques are not at the level required to enable this ultimate PHM vision; additional research is required.

### 2.2. Prognostics and Health Management for Smart Manufacturing Systems (PHM4SMS)

Within SMOPAC, the PHM4SMS project is aimed at developing the necessary measurement science to enable and enhance condition-monitoring, diagnostics, and prognostics. This measurement science includes the development of performance metrics, test methods, predictive modeling and simulation tools, reference data sets, protocols, and technical data.

The first of three phases of the PHM4SMS project is focused on assessment (the other phases are development and standardization): understanding the existing PHM capabilities, challenges, and needs of the manufacturing community and identifying the gaps that, if addressed, could benefit industry. This assessment phase has been marked by extensive research into PHM, both within literature reviews and direct interactions with PHM stakeholders (e.g., manufacturing process maintenance engineers, process design engineers, equipment operators). The NIST team has gained valuable insight about preventative/time-based maintenance (Ahmad & Kamaruddin, 2012) (Coats, Hassan, Goodman, Blechertas, Shin & Bayoumi, 2011); predictive maintenance/condition-based maintenance (Butcher, 2000) (Byington, Roemer, Kacprzymki & Galie, 2002) (Montgomery, Banjevic & Jardine, 2012) (Tian, Lin & Wu, 2012); and proactive/intelligent maintenance including maintenance at complex system levels (Barajas & Srinivasa, 2008) (Lee, Ghaffari & Elmeligy, 2011) (Lee, Ni, Djurdjanovic, Qiu & Liao, 2006).

Likewise, studies and reviews have been identified that compare existing PHM methods along with highlighting their strengths and limitations (Kothamasu, Huang & VerDuin, 2006) (Muller, Crespo Marquez & Iung, 2008) (Peng, Dong & Zuo, 2010). More specifically, reviews of PHM-based standards have also been conducted (Vogl, Weiss & Donmez, 2014a) (Vogl, Weiss & Donmez, 2014b) (Zhou, Bo & Wei, 2013).

Besides NIST efforts in reviewing the existing PHM techniques and standards landscapes, NIST has actively engaged numerous manufacturers to directly understand their PHM capabilities, successes, challenges, and needs. This has included site visits with many small, medium, and large manufacturers from a range of industries including automotive, aerospace, defense, earth-moving, and electromechanical. Stakeholder engagement peaked with the planning and execution of the *Roadmapping Workshop on Measurement Science for Prognostics and Health Management for Smart Manufacturing Systems*.

## 3. Workshop

The NIST PHM4SMS project team contracted with workshop facilitation and documentation experts at Energetics Corporation to host a two-day workshop. This workshop brought together PHM stakeholders including small, medium, and large manufacturers; technology developers and integrators; standards bodies; academic researchers; and U.S. government organizations. This section summarizes the workshop activities and the output information from the participants. The full details can be found in the comprehensive workshop report (National Institute of Standards and Technology, 2015a).

### 3.1. Goals

The workshop was planned and executed with three specific goals. They were to identify and prioritize the:

- Measurement science needs for improving PHM impacts within manufacturing processes;
- Measurement science barriers, challenges, and gaps that prevent the broad use of PHM technologies for manufacturing processes;
- R&D needed to address the priority measurement and standards challenges.

### 3.2. Plenary Talks and Panel Discussions

The workshop featured five plenary talks and three panel discussions (National Institute of Standards and Technology, 2015b). The plenary talks, presented by NIST personnel and external PHM experts, talked about the needs to evolve PHM technology within manufacturing along with existing PHM successes that several organizations have recently employed. Likewise, the talks highlighted specific challenges that still remain that, if addressed, can present tremendous benefit to the manufacturing community. These challenges included the development of common standards, interoperability among systems, deriving actionable intelligence from extensive data streams, and enabling machines to self-heal (i.e., impending faults or failures and automatically take corrective actions to remedy the problem).

The three panel sessions are discussed in the following subsections. Each panel was moderated, and included numerous speakers from diverse industry backgrounds, each with practical PHM experience. Some of the highlights from the question and answer sessions during each panel will be discussed herein. Full presentations given by both the plenary speakers and panelists can be found on the NIST web space (National Institute of Standards and Technology, 2015b).

#### 3.2.1. Panel 1: PHM Capabilities, Best Practices, Challenges, and Needs

This panel focused on the current state of PHM for manufacturing. Panelists focused on PHM technologies and systems including existing capabilities, best practices, and challenges along with technological gaps and limitations. Some of the key highlights of the panel’s question and answer session include:

- Communication and interoperability at the system level – Diversity, varying ages, and non-standard software of numerous systems add complexity to PHM systems. Enhancing, simplifying, and standardizing communications among multiple systems is warranted to streamline PHM.
- Catalog of data sets for understanding failure – It is challenging to obtain sufficient training data to ascertain when equipment or processes will fail. It is rarely practical to let a machine or process fail solely to obtain a realistic data set. Given that the best data is often from real failures, data must be opportunistically captured when a true fault or failure occurs.
- Real-time aspects of PHM technologies – Manufacturers are seeing an increasing need for real-time PHM technologies. This is especially true for high value equipment or processes where any faults or failures can be detrimental to overall manufacturing operations. Not all organizations are ready for this shift; some are still lacking in basic (not real-time) PHM while others do not see the implementation of real-time PHM as being cost effective for their operations.

#### 3.2.2. Panel 2: Performance Assessment – Monitoring and Measurement

This panel discussed the techniques for monitoring and measuring the performance of the PHM systems, themselves, along with identifying the metrics that evaluate how PHM technologies impact overall manufacturing performance. Highlights from this panel’s question and answer session include:

- Equipment monitoring and data collection by suppliers – It is challenging to implement PHM in one’s own organization and it can also be challenging to request PHM be integrated into an external supplier’s operations. Those suppliers that integrate PHM within their operations will likely gain a competitive advantage in that they will have more forewarning of faults and/or be more capable of handling unforeseen failures.
- Cost justification of PHM systems – When manufacturers buy manufacturing equipment that has a history of reliable operations, it is unlikely that they will also want to invest in PHM for this same equipment. The cost justification can be made in terms of maintaining or increasing quality and/or safety. Manufacturers will gain confidence in their equipment if PHM technology providers support any warranties that are tied to the equipment.

#### 3.2.3. Panel 3: PHM and the Human Element

The third panel focused on the influence and understanding of human decision-making on PHM systems within manufacturing and the difficulties that present themselves when humans work with PHM. A few of the highlights of this panel’s question and answer session include:

- Need for increased knowledge of refurbished equipment – It is difficult to accurately assess a machine’s health after it has been repaired (following a fault or failure), refurbished, or undergone extensive maintenance. This lack of knowledge can also complicate understanding a system’s overall health when a constituent component has been extensively repaired or replaced. This situation presents an opportunity to develop inventory tracking in conjunction with PHM that could document individual health states and expected remaining useful life (RUL) of specific components.
- PHM is easier to implement at the onset of a machine’s/process’ life – It is more cost effective and easier to integrate PHM into equipment or a process during the design stage prior to the equipment or process being put into service. This ease of implementation includes making it easier to integrate sensors, technology, and programming for PHM. One disadvantage of integrating PHM at the onset is that it is likely that all of the faults and failures that could/will occur are not known at this initial timeframe; some faults and failures are still likely to occur that the PHM system would either not detect or inaccurately detect. PHM design and implementation is costly, so the specificity and extent of its capabilities should be measured against the projected savings with its usage.

### 3.3. Breakout Sessions

The workshop featured three separate breakout topics: *PHM Manufacturing Process Techniques and Metrics, PHM Performance Assessment*; and *PHM Infrastructure* – *Hardware, Software, and Integration*. Each breakout topic met four times (Sessions I, II, III, and IV) across the two-day event and held a specific focus:

- Breakout Session I: Goals and Desired Capabilities – For each topic area, the first session focused on capturing the specific PHM capabilities most wanted and needed. Each group identified goals in the near-term (1 to 2 years), mid-term (3 to 5 years), and long-term (5+ years) time horizons. Additionally, each group then categorized the capabilities in the different topic areas in terms of high, medium, and low priorities.
- Breakout Session II: Challenges and Barriers for Achieving the Capabilities – This breakout meeting for each topic focused on identifying the specific measurement and standards barriers, challenges, and gaps that hinder PHM development, implementation, and integration.
- Breakout Session III: Prioritization of Challenges – This breakout meeting identified R&D and standards priorities for each of the challenges and barriers mentioned in the prior session. This included organizing the challenges in terms of high, medium, and low priorities.
- Breakout Session IV: Pathways for a Measurement Science Roadmap – The final breakout meeting organized each topic’s participants in small groups to develop specific roadmaps with recommended approaches, next steps, and actionable plans. Each action plan was also broken out into near-term (1 to 2 years), mid-term (3 to 5 years), and long-term (5+ years) timeframes.

Each of the three breakout topics will be presented in the following subsections. Although the three breakout groups operated separately, some of their identified goals, capabilities, challenges, and priority roadmaps had similar themes. This was natural in that some of these similarities cut across multiple topic areas. Cross-cutting themes are highlighted in Section 3.4.

In the following sections, highlights will be presented for all three breakout topics with a focus on the output roadmaps. Certain roadmaps were selected from each breakout topic for discussion in this paper. The chosen roadmaps were deemed the most important to address immediately and/or were supported by a majority of the participants while being relevant to NIST’s mission.

#### 3.3.1. Breakout Topic: PHM Manufacturing Process Techniques and Metrics

The successful implementation of PHM can have a significant influence on manufacturing operations by providing timely actionable intelligence. This intelligence can then be used to aid maintenance such that downtime is carefully coordinated with manufacturing operations for zero loss of productivity and quality. This breakout topic focused on addressing the specific PHM manufacturing process capabilities that can enable this timely actionable intelligence along with the metrics necessary to collect and analyze in support of these capabilities. This group focused on PHM techniques and metrics that can ultimately enhance condition-monitoring, equipment and process reliability, safety, operator situational awareness, and overall equipment effectiveness. After the group identified their desired goals and capabilities, and the corresponding challenges and barriers, three priority roadmap topics were developed. Two of the roadmaps are presented below while the third (Enterprise-Wide PHM for Maintenance Planning) is not discussed due to space restrictions.

##### Advanced Sensors for PHM in Smart Manufacturing

The development of this specific roadmap was spurred by the lack of understanding of the full suite of capabilities of sensors, their interfaces and interoperability needs for PHM. This is critical to address because current PHM systems lack re-configurability, flexibility, scalability, and robustness partly due to the lack of knowledge with respect to sensors.

A sub-group within this breakout session focused on outlining a multi-stage method for sensor development. This approach begins by inventorying existing sensor data acquisition (DAQ) systems that are needed for PHM systems and defining the re-configurability requirements for common manufacturing processes. This effort would ultimately breed data communications and analytics standards to promote greater communication among multiple configurations and technologies. Mid-term activities would include the identification of gaps in sensor and DAQ capability and interoperability, and define scalability requirements for several manufacturing processes. Long-term activities feature the development of multi-purpose sensors/DAQ interfaces for use within manufacturing PHM systems; development of standards for data communication, data analysis, and prognostic algorithms; and the development of a taxonomy of PHM systems and capabilities. This would lead to the generation of a taxonomy library and a PHM-handling catalog of generated tools to promote flexible and reconfigurable PHM systems. The completion of this roadmap action plan is envisioned to have high impact within the manufacturing community since it’s very likely to improve reliability/reduce failures of equipment and processes, improve maintenance scheduling, and speed process reconfigurability.

##### PHM Data Format and Architecture

The generation of this roadmap was motivated by the desire to solve the lack of interoperability of sensors/data formats and types of communication while preserving the meaning of the data and the semantics. The overall approach of this roadmap is to create protocols for PHM covering formats, storage, organization, semantics, and other key components. Standards would be created to support the protocols along with data interfaces and integration. These protocols and overall architecture would enable the generation of a database of PHM data and information that the community could draw upon.

The near-term activities of this roadmap include determining protocol data types and structure. Guidelines must also be developed for data format, storage and preservation, organization, and semantic requirements. Moving forward, the mid-term activities would focus on standards development for semantic PHM data and the creation of tools to capture and organize the data; and then, extract and visualize the information in a meaningful way. The long-term tasking would focus on the creation, organization, and management of PHM data repositories. This would yield an expansive database that could be used by manufacturers, technology integrators, and technology developers who work with PHM systems. The advancement of this roadmap would have the highest impacts in speeding process re-configurability and improving maintenance scheduling.

#### 3.3.2. Breakout Topic: PHM Performance Assessment

Before any new technology can realize its full potential, it is critical to verify and validate its performance. PHM is no exception, and care must be taken to ensure that any PHM technology’s performance and impact is accurately assessed. This breakout topic focused on assessing the performance of PHM along with the necessary technologies, measurement techniques, data, and performance metrics required for such verification and validation.

Breakout participants identified goals in the areas of identifying specific PHM performance characteristics and metrics, and equipment and technologies necessary to monitor a PHM system (or component). In addition, the participants also noted the long-term goal of incorporating the design and validation of a PHM system into the overall equipment/process life cycle. Once the participants identified the subsequent capabilities and existing challenges, six priority roadmap topics were developed. Three of the roadmaps are presented in detail while the remaining three (Cost Model for PHM Performance, Taxonomy of Applications, and Determination of PHM Data and Information Needs) are not presented due to space restrictions.

##### Overarching Architecture Framework for PHM with Standards and Key Performance Indicators (KPIs)

This roadmap was motivated by the participants’ acknowledgement that a PHM framework within multiple industries is either unclear or lacking in standards. This absence of standards promotes inconsistencies in PHM verification and validation.

The ultimate goal of this roadmap is to define a standard PHM architecture and create methods that will enable asset traceability and historical record keeping on PHM performance. To realize this goal, the participants identified the near-term action of benchmarking the current state of machine monitoring (starting with specific industries) and the mid-term tasks of cataloging the KPIs and mapping-out the typical diagnostic and prognostic trends (from the target industries). The vision is that this effort would produce a published catalog that gains some industry acceptance (100% acceptance is too ambitious at this time, yet an initial target was not determined). Likewise, international standards would be developed that are broad enough to cover a range of PHM implementations across multiple industries. These standards would have to be specific enough to guide manufacturers through the process of developing and implementing a means of verifying and validating their PHM capabilities. If successful, this roadmap is expected to have significant impact in improving equipment/process reliability, reducing costs, increasing industry’s competitiveness, and enhancing maintenance scheduling.

##### Identification of PHM Performance Metrics

Participants produced this roadmap citing a lack of performance metrics capable of characterizing the value of prognostics to equipment or processes prior to failure. This coincides with limited information on key metrics for manufacturing equipment and/or processes at component and system levels. The overall approach proposed is to evaluate existing metrics to determine what metrics can be captured from equipment/processes prior to a fault or failure that sufficiently evaluate the performance of the PHM system in question. This assessment will aid in developing new performance metrics.

The near-term plans of this roadmap feature three activities: 1) survey current metrics that characterize the performance of a PHM system itself and the PHM’s effectiveness when applied to a machine/process, 2) identify the necessary metrics that can apply diagnostics and prognostics to manufacturing equipment/process and integrate with controls/operations and maintenance planning, and 3) determine the gaps present between existing and desired metrics. Mid-term actions include 1) developing the missing metrics, 2) evaluating the metrics across a range of equipment, processes, systems, and PHM algorithms, and 3) studying how performance metrics can be integrated with controls, operations, and maintenance planning systems. Long-term activities conclude with integrating the identified performance metrics with the PHM architecture (described in the prior section) so the metrics can be implemented and demonstrating the applied metrics (in concert with the architecture) at selected pilot plants. The achievement of implementing the metrics and framework in a plant is envisioned to be a stepping-stone to applying the metrics and framework to additional manufacturing facilities.

The expected impact of completing this roadmap action plan includes better decisions being made based upon available PHM results and performance metrics; improved quality and productivity of equipment and processes; and greater availability of actionable information.

##### Failure Data for Prognostics and Diagnostics

The final roadmap is motivated by the lack of sufficient, available failure data for diagnostics and prognostics. Currently, measurement and data collection methods and appropriate test beds are limited in their availability and capability. For those methods and test beds that do exist, there is a lack of consistency in the data formats for which data is captured and organized. The participants who developed this roadmap proposed the approach of developing methods and services to generate diagnostic and prognostic data sets for public use including verification and validation. This would be supported by the development of specific test beds that would enable both the production of data and the necessary verification and validation.

The roadmap action plan begins with three near-term activities: 1) development of a common database, 2) creation of test beds to assess feasibility, and 3) establishment of a consortium (including NIST and university partners) to examine PHM for specific systems in the form of test bed(s). Mid-term activities include qualifying the data within the common database and further development of the scaled-down test beds. Long-term activities feature the implementation and testing of the common database, standardizing the scaled-down test beds, and performing simulation modeling of processes. Upon the completion of these tasks, the realized capabilities should be the active use of a common database and the adoption of PHM failure data standards. The realized impact of these capabilities is expected to include a significant reduction in cost (this method promotes cost sharing across the industry) and improved access to failure data to support verification and validation of PHM methods.

#### 3.3.3. Breakout Topic: PHM Infrastructure – Hardware, Software, and Integration

Successful PHM methods and technologies require a robust infrastructure including key building blocks such as hardware, software, models, and simulations along with the integration of these elements. Technology has greatly advanced in the last decade (including enhanced capabilities in wireless connectivity, mobile devices, computing power, sensing capability, and human machine interfaces), and the PHM infrastructure has become increasingly complex. The participants in this breakout topic discussed a variety of infrastructure needs from the perspective of enabling and augmenting PHM within smart manufacturing environments.

Breakout participants identified near-term, mid-term, and long-term infrastructure goals in the areas of PHM design, hardware, software, security, maintenance, and data management. This prompted the participants to identify the capabilities and their corresponding priorities. Next, the participants identified the challenges and barriers to achieving these capabilities and prioritized them accordingly. These efforts led to the development of four roadmap action plans. Two of the roadmaps are presented in detail while the remaining two (PHM as an Equipment Design Feature and Embedded Sensors for PHM of Emerging Manufacturing Technologies) are not discussed due to space restrictions. Those roadmaps not discussed in this paper can be found in detail in the full workshop report (National Institute of Standards and Technology, 2015a).

##### Open-Source Community for PHM

The first roadmap action plan to be presented from this breakout topic is motivated by the fact that it is often costly and overly complex to implement PHM on new equipment. The proposed approach charts the path of developing an open source architecture that will reduce the cost and complexity of PHM design and implementation. The approach features a collection of data and identification of relevant PHM systems and devices.

The near-term activities of this roadmap include: 1) the development of open drivers and adapters enabling PHM through the integration of sensors, equipment, controllers, interfaces, etc. 2) the expansion of the data collection infrastructure to accommodate an open source format, and 3) the development of security, compression, fault tolerance, and schema for the open architecture. Mid-term tasks include: 1) identification of systems and devices to be compatible with the framework, 2) development of frameworks and toolkits to enable users to interface with equipment, and 3) expansion of drivers and adapters. Finally, the long-term task is a continuation of the prior tasks – promote continuous development and improvement (similar to what is done in the Linux community). The goal is to get a majority (ideally, all) of industry (ranging from small to large enterprises) using and contributing to the open architecture.

If this roadmap action plan is successfully completed, numerous impacts could be realized. The most significant impacts that could be realized include reduced individual cost to develop and implement PHM; accelerated pace of innovation since more time could be devoted to developing PHM algorithms as opposed to developing the architecture (since it would already be in place); and enhanced industrial competitiveness since the increased presence of PHM would reduce maintenance costs and enhance versatility.

##### PHM Infrastructure to Deliver Relevant Timely Information

The final roadmap action plan to be presented is similar to the roadmap highlighted in the last section, yet is still unique in scope and objectives. The participants developed this plan to overcome the current inability to make good decisions based upon the available data where PHM users are currently making decisions either with the wrong information, with insufficient detail, and/or at the wrong levels. The proposed approach focuses on developing a traffic light approach (e.g., green, yellow, red) to classifying the value of the data for decision-making.

This roadmap features an extensive action plan with eight near-term and six mid-term tasks identified. Some of the near-term tasks include the development of tools to construct cyber-models of replacement parts/components to better predict RUL or mean time to failure, determination of required data to model diagnostics and prognostics, and assess requirements to determine the necessary information needed at each operational level within a manufacturing environment. Several of the mid-term tasks include the development of a cloud-based data repository and analytic engine to further enhance decision-making and technology generation to enable adaptable alarms based upon equipment/process condition. The participants identified a single long-term goal – develop advanced usage-based models to augment PHM decision-making. Increased and enhanced decision-making is the ultimate desired capability where the participants envision 80% improvement (over existing baselines) after five years of effort on this roadmap.

The significant impacts that could potentially be realized if this action plan is completed are the generation and availability of better data for fault and failure prevention, and appropriate data and better decision-making are fused to make timely decisions regarding maintenance scheduling.

### 3.4. Cross-Cutting Themes

Over the entire course of the workshop, numerous themes emerged, both within the individual breakout topics and across the rest of the workshop program (plenary talks and panel discussions). Six specific themes were identified; three are presented in the following sub-sections while the other three (Workforce and Training, Human Factors, and Business Case for PHM) are not presented.

#### 3.4.1. Data Collection and Extraction of Information

The challenges of collecting, extracting, and analyzing appropriate and meaningful data were well documented throughout the workshop. Data is a critical piece of designing, verifying, validating, and implementing effective PHM technologies into a manufacturing process or piece of equipment. These challenges stem from a lack of sensors capable of capturing the right data at the appropriate frequency, accuracy, and resolution; and a lack of rigorous measurement methods to enable efficient and effective data collection methods suited for PHM. Additionally, inconsistent or insufficient data standards are making it difficult to broadly apply PHM across a range of manufacturing equipment and processes; standardization of data formats and taxonomies would play a significant role in overcoming this challenge. Another data challenge is generating accurate PHM data, for the purposes of PHM design, verification, and validation without damaging equipment or decreasing productivity.

#### 3.4.2. Models, Simulation, and Visualization

Validated models to support PHM are limited in availability and capability. The entire scope of modeling, simulation, and visualization (MSV) is also encumbered by the diversity of manufacturing equipment and processes, lack of integration with legacy systems, and data availability (which is critical for effective MSV). A benefit of having accurate and relevant models is that they can help highlight the value of PHM prior to a system being put into practice. This would help generate further organizational support for PHM, and it sets initial expectations of the predicted performance.

#### 3.4.3. Design Considerations

The last cross-cutting theme to be highlighted is the notion that PHM be considered as a design feature that is factored in to the design process of any new piece of manufacturing equipment or process. Most original equipment manufacturers (OEMs) do not consider PHM in their design process; any PHM that is factored typically include limited forms of condition-monitoring and diagnostics. Likewise, most technology integrators will not add PHM into their process design unless their customer specifically requests PHM and is willing to pay the additional costs for it. It is much more challenging to integrate effective PHM into a system/process after that system/process is in service on a factory floor.

## 4. NIST’s Research Direction

The workshop provided valuable insight that is envisioned to bring tremendous benefit to the PHM community. Likewise, NIST is carefully reviewing the workshop findings to update its project’s research direction to further align it with industry’s needs and high priorities. The PHM4SMS project team is currently focused on four specific efforts that are all factoring in the workshop findings.

### 4.1. Machine Tool Linear Axes Diagnostics

This effort is focused on developing a sensor-based method to quickly estimate the degradation of linear axes, and is supported by the development of a linear axes test bed. This method leverages data collected from a NIST-developed sensor suite to detect translational and angular changes due to axis degradation. Real-time data is collected to enable diagnostics and prognostics of linear axes for optimization of maintenance scheduling and part quality. This method to estimate the degradation of linear axes will also enable verification and validation of other (built-in or otherwise) PHM techniques that aim to characterize translation and angular errors and degradation. Likewise, this method will produce reference data sets that can be used by PHM developers as test data so they do not have to risk damaging their own equipment or impacting their productivity. This method will ultimately lead to standards to measure and predict linear axes degradation. The linear axes test bed will yield its first data sets for analysis in Summer 2015.

### 4.2. Manufacturing Process and Equipment Monitoring

Driven by the need to identify high-value data sources and the most appropriate times to collect data, this manufacturing process and equipment monitoring effort focuses on enabling the seamless and effective use of data to generate timely and actionable intelligence on equipment/process health. This effort is supported by the development of a systems-level test bed of networked machine tools and sensors in an active manufacturing facility. Accordingly, a significant part of this research is the design of a reference implementation that manufacturers may use to collect data safely and efficiently without disruption to operations. Likewise, this effort will also yield a reference dataset of fabrication and inspection data that may be used to identify useful links for improved process monitoring, diagnostic, and prognostic capabilities. This test bed will produce initial results in Fall 2015.

### 4.3. Systems-Level Diagnostics and Prognostics

Many complex processes and systems-of-systems are lacking in higher-level capabilities to accurately and efficiently forecast faults and failures. This research effort addresses this challenge by developing protocols to communicate data, information and metrics across the component, sub-system, and system levels for diagnostics and prognostics in manufacturing. These protocols will enable the prediction of system-level impacts of events occurring at a single component or sub-system. Moreover, the protocols will enable and enhance process management and control approaches to effectively respond to these events. A hierarchical methodology is being developed with external partners, and will be applied to the two aforementioned test beds in 2016.

### 4.4. PHM for Robotics

Robotics are increasing in their implementation and complexity of integration within manufacturing operations. PHM considerations of a robotic system extend beyond just the physical arm, gantry, mobile base, etc. nearly every robotic system features some type of end-effector, sensors, safety system(s), supporting/surrounding automation, controller, etc. Robotic systems, especially in smart manufacturing environments, are often marked by complex interactions among these elements. For example, a fault or failure that presents itself as unexpected or inappropriate behavior of the robot arm is likely to have resulted not from a mechanical failure of the arm, but rather from a failure elsewhere in the system (e.g., sensor failure, or a controller fault). This research effort is actively developing a PHM-focused robotics test bed that features a scaled-down industrial robotic arm system to develop test methods, metrics, assessment protocols, and reference data sets that can evaluate robot system degradation techniques including how such degradation impacts key elements of the robot system (e.g., safety). This test bed is expected to be operational and produce its first data sets in Summer 2016.

## 5. Conclusion

The two-day workshop brought together many PHM experts who shared their best practices, challenges, and visions with respect to PHM in smart manufacturing (National Institute of Standards and Technology, 2015a). Their extensive feedback is well-documented in the roadmap action plans, and will guide the community in devising and updating their research directions, accordingly. As a member of the community, NIST is examining the workshop findings to best determine where its research efforts can have substantial impact in addressing PHM measurement science challenges.
